# Tailoring Perpendicular Exchange Bias Coupling in Au/Co/NiO Systems by Ion Bombardment

**DOI:** 10.3390/nano8100813

**Published:** 2018-10-10

**Authors:** Piotr Kuświk, Alexander Gaul, Maciej Urbaniak, Marek Schmidt, Jacek Aleksiejew, Arno Ehresmann, Feliks Stobiecki

**Affiliations:** 1 Institute of Molecular Physics, Polish Academy of Sciences, 1760179 Poznań, Poland; urbaniak@ifmpan.poznan.pl (M.U.); marek.schmidt@ifmpan.poznan.pl (M.S.); jacek.aleksiejew@ifmpan.poznan.pl (J.A.); stfeliks@ifmpan.poznan.pl (F.S.); 2 Institute of Physics and Center for Interdisciplinary Nanostructure Science and Technology (CINSaT), University of Kassel, 34125 Kassel, Germany; gaul@uni-kassel.de (A.G.); ehresmann@physik.uni-kassel.de (A.E.)

**Keywords:** exchange bias, perpendicular magnetic anisotropy, ion bombardment

## Abstract

Here, we systematically investigated the influence of ion bombardment with different fluences on the strength and direction of the exchange bias coupling in Au/Co/NiO systems with perpendicular magnetic anisotropy of the Co layer. We found that the direction of the exchange bias coupling can be reversed as a result of ion bombardment performed in an external magnetic field which is in the opposite direction to the magnetic field applied during film deposition. Moreover, the strength of the exchange bias coupling can be tailored by varying the ion fluence. These results show behaviors similar to the results found for systems of ferromagnetic layers with in-plane anisotropy. Our experimental work, supported by a two-energy-level model, demonstrates that exchange bias coupling can be tuned in a layered system with perpendicular magnetic anisotropy using ion bombardment.

## 1. Introduction

The Exchange-Bias (EB) effect in antiferromagnet (AFM)/ferromagnet (FM) thin film systems with in-plane [1,2,3,4] and perpendicular magnetic anisotropy (PMA) [5,6] is well known. In such systems, the direction of the exchange bias is initialized by field growth (layer deposition in an external magnetic field (*H*_dep_) [1]), by field cooling (FC) (heating and subsequent cooling of the layer system through the ordering temperature of the AFM layer in the presence of an applied magnetic field [7]), by thermally-induced ordering (annealing of AFM alloys to create the required AFM order [8]), or by light-ion bombardment-induced EB initialization (bombardment of the AFM/FM layer system by light ions in an external magnetic field (*H*_IB_) [9]. The latter method has additionally been used to tailor the unidirectional anisotropy after its initialization in thin film systems with in-plane unidirectional anisotropies [10,11,12,13,14]. In such systems, depending on the ion fluence (*F*), the strengths of EB can be either increased or decreased, whereas its direction can be tuned by the direction of the external magnetic field *H*_IB_.

Apart from in-plane EB layer systems, light-ion bombardment has been used to tune the perpendicular magnetic anisotropy in multilayer films [15,16,17,18], a technology successfully used to fabricate magnetic patterns of individually switchable monodomain areas [19]. Up to now, however, there is no information on whether it is possible to tailor EB layer systems with perpendicular unidirectional anisotropy by ion bombardment.

Here, we report on ion bombardment of a thin film system with perpendicular EB, with and without an external magnetic field. In both cases we show that the perpendicular EB field can be tailored both in magnitude and in direction by changing the ion fluence and the direction of *H*_IB,_ respectively.

## 2. Materials and Methods 

For the ion bombardment experiments, two multilayer (ML) systems (Ti 4 nm/Au 60 nm/Co-wedge 0–2 nm/NiO10 nm/Au2 nm and Ti 4 nm/Au 60 nm/Co 0.6 nm/NiO 10 nm) have been deposited onto naturally oxidized Si(100) substrates in a multi-chamber system with base pressure below 5 × 10^−8^ mbar. The Ti, Au, and Co layers were deposited by magnetron sputtering in an Ar atmosphere (1.4 × 10^−3^ mbar) and the NiO layer was deposited using pulsed laser deposition in an oxygen atmosphere (1.5 × 10^−5^ mbar) for proper stoichiometry [20,21]. For deposition an ultra-pure Ti (Testbourne Ltd., Basingstoke, UK), Au (Mennica Metale Szlachetne S.A., Warsaw, Poland), Co (Kurt J. Lesker Company Ltd., Hastings, UK), and NiO (MaTeck GmbH, Jülich, Germany) targets were used. The deposition rate for Co, Ti, Au, and NiO are 0.45, 0.39, 1.28, and 1.59 /s, respectively. The deposition rate was calibrated by two independent techniques, X-ray reflectivity and quartz balance. The Co wedge layer was realized by moving a shutter with constant velocity during Co deposition. To initialize the EB between the Co and the NiO layers, the samples were deposited in a magnetic field *H*_dep_ oriented perpendicularly to the sample plane.

Ion bombardment has been performed with fluences specified further in the text by a commercial Focused Ion Beam (FIB) (FEI, Hillsboro, OR, USA) system with 30 keV Ga^+^ ions and with 10 keV He^+^ ions from a home-built plasma source [22]. After the ion currents were measured using a Faraday cup, the exposure times were selected to obtain the desired the ion fluence. Both processes were performed in configuration, in which the ion beam was perpendicular to the sample plane. To modify the exchange bias field, the 10 keV He^+^ ion bombardment was carried out in an external out-of-plane magnetic field *H*_IB_ = 1.1 kOe generated by the permanent magnet.

Local hysteresis loops for the sample with the wedge layer were measured at different positions along the wedge using a home-built polar magneto-optical Kerr effect (P-MOKE) magnetometer with a laser spot diameter of 0.4 mm. Images of magnetic domains for the sample with layers of constant thicknesses were recorded by a P-MOKE wide-field microscope (Carl Zeis, Germany) at 100× and 200× magnifications. All magnetic measurements were performed ex situ at room temperature.

## 3. Results

**Figure 1:**
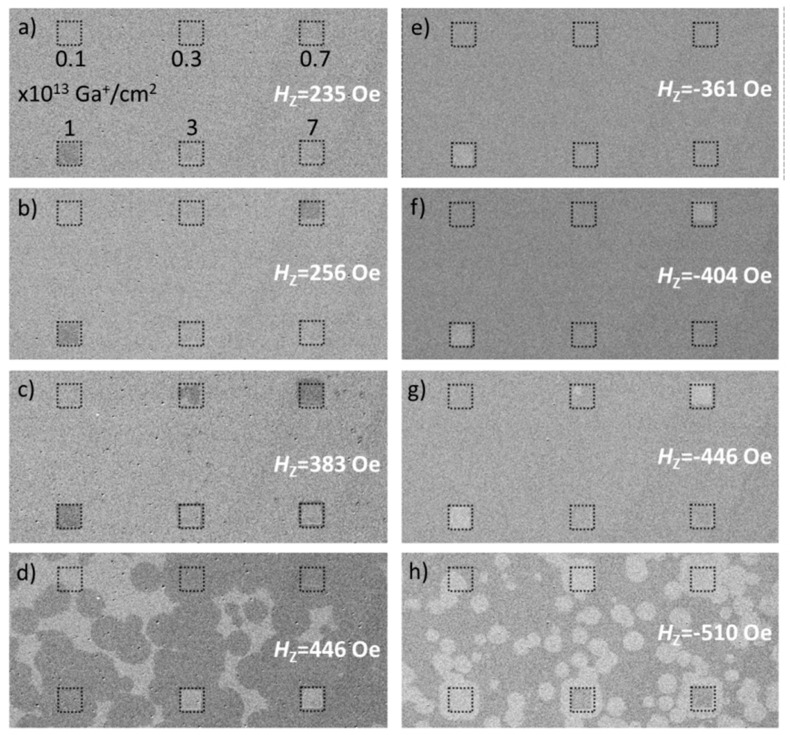
Differential polar magneto-optical Kerr effect (P-MOKE) images of the domain structure recorded in remanence for non-bombarded areas and for areas bombarded with different fluency F (dotted squares), taken after different *H*_z_ pulses (**a**–**h**). Prior to the measurements, the Ti 4 nm/Au 60 nm/Co 0.6 nm/NiO 10 nm sample was saturated at *H*_z_ = −2100 Oe (**a**–**d**) and at *H*_z_ = +2100 Oe (**e**–**h**).

In the first part of the paper, we will describe the influence of 30 keV Ga^+^ ion bombardment within a wide *F* range of 0.1–7 × 10^13^ Ga^+^/cm^2^, without applied magnetic field during the bombardment, on the magnetic properties of the Au 60 nm/Co 0.6 nm/NiO 10 nm ML. The ion bombardment was realized across 50 × 50 µm^2^ areas using FIB. The modified areas were chosen large enough to study the influence of different *F* on the magnetic structure by P-MOKE microscopy (Figure 1), and their distances were chosen large enough to exclude interactions between the modified areas. The domain patterns have been observed at remanence by P-MOKE microscopy as functions of the strengths of perpendicularly-oriented magnetic field pulses in two opposite directions (±*H*_z_), applied for 1 s, following the magnetic saturation of the layer system by a magnetic field oriented antiparallel to the field pulse direction. This enables us to determine the switching fields for the two opposite directions of *H*_z_ ($H_{s}^{+}$ and $H_{s}^{-}$) necessary to calculate the coercivity ($H_{C}=\frac{\left|H_{s}^{+}\;\left|+\right|H_{s}^{-}\;\right|}{2}$) and exchange bias field ($H_{EB}=\frac{H_{s}^{+}+H_{s}^{-}}{2}$). In this sample, EB was induced by deposition of the layer system in an external magnetic field of *H*_dep_ = 1.1 kOe.

**Figure 2:**
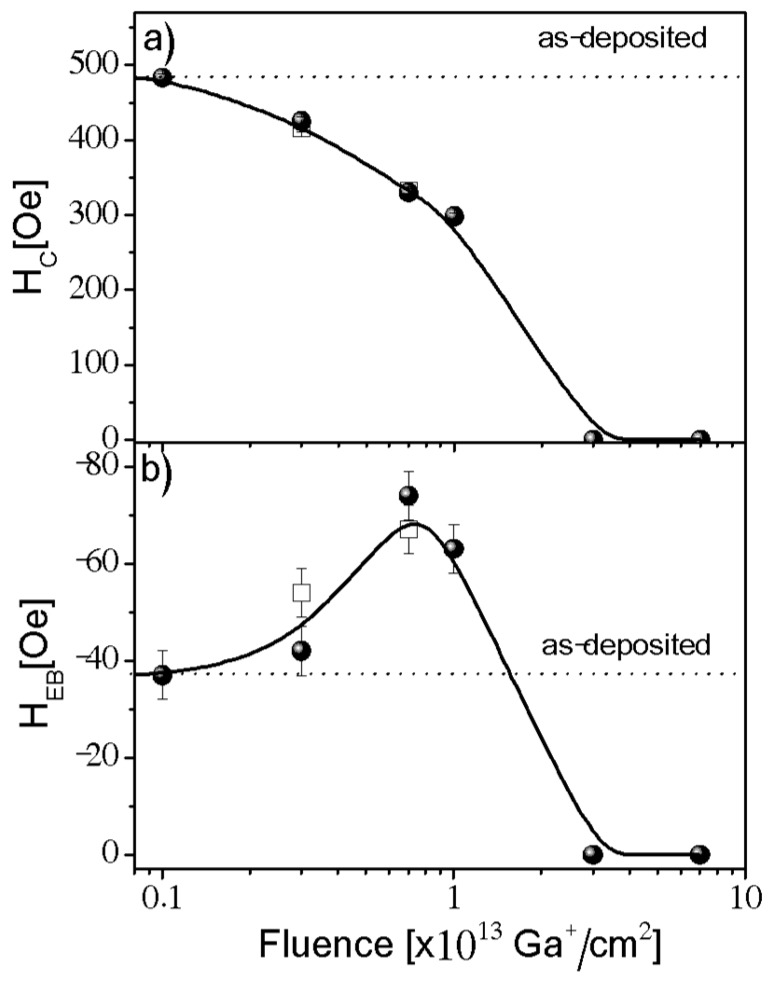
(**a**) Coercivity (*H*_C_) and (**b**) exchange bias field (*H*_EB_) as functions of the 30 keV Ga^+^ ion fluence, determined from the P-MOKE images recorded at 100× (full circle) and 200× (open squares) magnifications. Values for the as-deposited samples are shown by the horizontal dotted lines. The solid lines are guided to the eye.

For the smallest fluence, *F* = 0.1 × 10^13^ Ga^+^/cm^2^, the ion bombardment produced no observable changes in the magnetic pattern of the sample surface. This is in good agreement with recent reports [23,24] indicating that exceeding a fluence threshold is necessary to induce a macroscopically-relevant modification of magnetic properties by ion bombardment. Significant changes appear for 0.3 *F* 1 × 10^13^ Ga^+^/cm^2^, where *H*_C_ distinctly decreases but PMA is preserved. Simultaneously, an increase of *H*_EB_ is observed (Figure 2). For the two largest investigated fluencies of *F* 3 × 10^13^ Ga^+^/cm^2^, no changes in the magnetic contrast with *H*_z_ were observed in the P-MOKE images. This suggests that these fluencies destroy the PMA [25], or the Co layer is transformed into a non-ferromagnetic state because of mixing with surroundings layers. This is consistent with previous results for NiFe/Au/Co/Au [24]. Comparing the observed changes of *H*_C_(*F*) with those previously reported [23] for other PMA systems, we can conclude that it is a typical relationship, which matches results for Pt/Co/Pt bombarded by 30 keV Ga+ ions. The *H*_EB_(*F*) dependence shows a similar behavior to that observed for EB thin-film systems with in-plane anisotropy (e.g., IrMn/Co [26], NiO/NiFe [27]). Unfortunately, due to limitations imposed by the FIB chamber, we were not able to perform Ga^+^ ion bombardment assisted by a magnetic field to verify the ability to change its sign, as was demonstrated recently [26,28].

**Figure 3:**
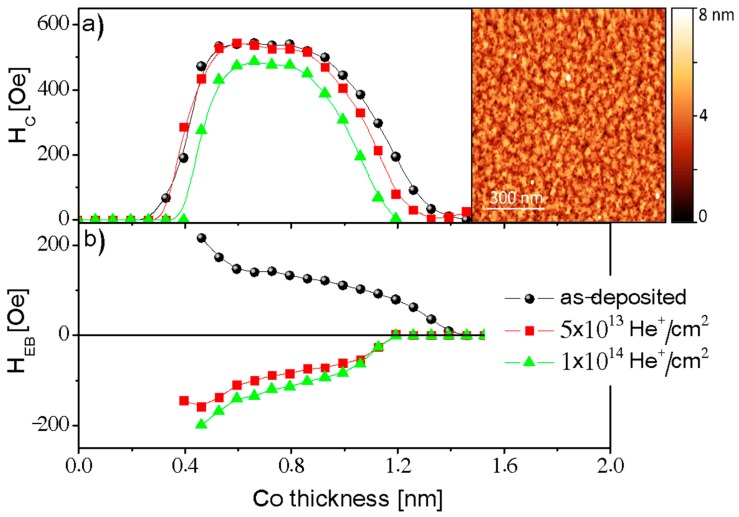
(**a**) Coercivity (*H*_C_) and (**b**) exchange bias field (*H*_EB_) for the Ti/Au/Co-wedge/NiO/Au sample with perpendicular magnetic anisotropy, before and after 10 keV He^+^ ion bombardment in an external magnetic field (*H*_IB_) applied in the direction opposite to *H*_dep_. The solid lines are guided to the eye. The inset shows the topography of the Ti/Au/Co/NiO/Au sample registered using atomic force microscopy.

To verify the role of the external magnetic field applied during ion bombardment in determining the direction of the exchange bias field in layered systems with PMA, we performed 10 keV He^+^ ion bombardment with an applied field *H*_IB_ (=1.1 kOe) oriented opposite to the direction of *H*_dep_ (= −1.1 kOe) for three different ion fluencies: 5 × 10^13^, 10^14^, and 10^15^ He^+^/cm^2^. Additionally, the influence of ion bombardment has been studied as a function of Co layer thickness using the Ti4 nm/Au60 nm/Co-*t*_Co_/NiO 10 nm layered system with the wedge-shaped Co layer (0 *t*_Co_ 2 nm). When the ion bombardment is performed with *H*_IB_ applied antiparallel to *H*_dep_, *H*_EB_ can be reversed (Figure 3) and its strength depends on the ion fluence. In a way similar to the results for low ion fluency Ga^+^ ion bombardment, the value of *H*_C_ remains almost unchanged and *H*_EB_ decreases slightly. Most importantly, however, is that after bombardment in an opposite field, *H*_EB_ changes sign. An increase in the fluence from 5 × 10^13^ to 10^14^ He^+^/cm^2^ results in two effects: (i) the range of thicknesses that preserve PMA is reduced, and (ii) the magnitude of the reversed exchange bias is increased. For *F* = 10^15^ He^+^/cm^2^, the perpendicular magnetic anisotropy is destroyed for all Co thicknesses, which is in good agreement with our previous results for the NiFe/Au/Co/Au system [18,24].

## 4. Discussion

The FIB experiments clearly show that *H*_EB_ can be enhanced by ion bombardment. This can be explained by the existing model for in-plane anisotropy polycrystalline layer systems [10,29,30]. Briefly, a two-energy-level model [29] can be applied to describe the coupling for a single domain AFM grain in contact with the FM layer. Note that, in our case, the NiO layer shows a polycrystalline structure with a grain size below 100 nm (inset in Figure 3). Therefore, we assume that each AFM grain has a single domain structure. The free energy of a system with uniaxial and unidirectional anisotropy for each AFM grain is described by $E=K_{AF}Vsin^{2}\alpha -J_{EB}Scos\alpha$, where *K*_AF_ is the antiferromagnetic anisotropy constant of the AFM grain with effective volume *V*, *S* denotes the interface area coupled with the FM at the FM/AFM interface with effective exchange coupling constant *J*_EB_, α is the angle between the FM magnetization direction (in our case perpendicular to the sample plane) and the pinned uncompensated AFM interface moment. We expect that the deposition of the NiO layer in *H*_dep_, which is oriented perpendicular to the sample plane, increases the population of out-of-plane AFM domains. As was found previously, the magnetic field applied during the FC process and the effective field from the FM layer may reorient NiO spins along their directions [31,32,33].

In this approach, two energy minima (local and global) appear for certain classes of grains (classes III and IV of reference [34]) as a function of α, separated by an energy barrier Δ (see Figure 3 in Reference [8]). This model describes well the exchange bias effect in polycrystalline systems, where AFM grains are too small to support multiple domains. In such a case, the *H*_EB_ is proportional to the difference between the number of AFM grains (domains) in the global minima and those in the local minima for grains with “temporally stable” coupling. The definition of “temporally stable” depends on the ratio of the grain transition time τ between the two minima and the operational process times (e.g., for storage between EB initialization and the duration of magnetic characterization). Additionally, the existence of grains with rotatable anisotropy may contribute to the change of *H*_EB_. To modify *H*_EB_ in these cases, the energy barrier Δ has to be surpassed by supplying the necessary excitation energy. This energy is typically supplied by heating or by the electronic energy loss of ions traveling through the layer system.

It was shown that application of FC procedure increases the *H*_EB_ in comparison to *H*_EB_ obtained after deposition at *H*_dep_. Therefore, in some cases, the EB is initialized by the magnetic field applied during film growth and afterwards is stabilized by FC [27]. In our case, the EB between the Co and NiO layers was established only by deposition in *H*_dep_. Therefore, we expect a stronger modification of the *H*_EB_ by ion bombardment than the one for an in-plane system, where the EB was induced after FC. In this case the maximum *H*_EB_(*F*)/*H*_EB_ (*F* = 0) is around 1.5 [10,35], whereas in our case it is about 2 (Figure 2b).

For 10 keV He^+^ ion bombardment under the *H*_IB_ antiparallel to *H*_dep_, the *H*_EB_ changes its sign. Note that in a sufficiently strong perpendicular external magnetic field (here *H*_IB_ = 1.1 kOe, which is much higher than the coercivity of the Co layer), the magnetization direction of the FM layer is aligned with field’s direction. As a result, the energy landscape described by the two-energy-level model is reversed because in the local (global) minima the uncompensated moments of the AFM domains are aligned antiparallel (parallel) to the magnetization direction of the FM layer, keeping the same populations of the AFM domain in each minimum. Ions traveling in the layered system cause the local hyperthermal energy transfer [10] mainly by deposition of energy (*E*) via electronic energy losses, which is high enough (e.g., the dE/dz equals to 9.48 eV/0.1 nm for 10 keV He^+^ ions bombardment of the NiO layer [36]) to overcome the barrier Δ. As a result, the sign of the perpendicular EB is reversed (Figure 3) and *H*_EB_ is smaller for *F* = 5 × 10^13^ He^+^/cm^2^ than for *F* = 10^14^ He^+^/cm^2^, which shows that EB increases in a way similar to what was presented for FIB bombardment described above.

## 5. Conclusions

We found that when ion bombardment is performed with *H*_IB_ applied antiparallel to *H*_dep_, the perpendicular EB can be reversed and its strength tailored by the application of ion fluence and an external magnetic field during bombardment, similar to what was described for in-plane anisotropy systems. Independently, we have shown that the Focused Ion Beam can be also used for manipulation of perpendicular EB. In particular, the strengths of EB can be enhanced by 30 keV ions in a fluence range between 3 × 10^12^ and 10^13^ Ga^+^/cm^2^. All this opens a new way to control magnetic properties or to create artificial magnetic structures on the basis of polycrystalline EB systems with PMA.
